# Validity and reliability of the Berlin questionnaire for the detection of moderate or severe obstructive sleep apnea in patients aged 40 years or older detected from primary care

**DOI:** 10.3389/fmed.2023.1229972

**Published:** 2023-08-14

**Authors:** Esther Navarrete-Martínez, Rafaela Muñoz-Gómez, Jesús Serrano-Merino, Luis Angel Perula-de Torres, Manuel Vaquero-Abellán, Fátima Silva-Gil, Ana Roldán-Villalobos, Enrique Martín-Rioboó, Javier Ruiz-Moruno, Esperanza Romero-Rodríguez, Jesús González-Lama, Gertrudis Montes-Redondo

**Affiliations:** ^1^Marchena Health Center, Osuna Sanitary Area, Seville, Spain; ^2^Maimonides Institute for Biomedical Research of Córdoba (IMIBIC)/Reina Sofía Hospital/University of Córdoba, Córdoba, Spain; ^3^Sector Sur Health Center, Córdoba-Guadalquivir Health District, Córdoba, Spain; ^4^Program of Preventive Activities and Health Promotion (PAPPS), semFYC, Barcelona, Spain; ^5^Faculty of Medicine and Nursing, University of Córdoba, Córdoba, Spain; ^6^Pedro Abad Health Center, Córdoba-Guadalquivir Health District, Córdoba, Spain; ^7^Carlos Castilla del Pino Health Center, Córdoba-Guadalquivir Health District, Córdoba, Spain; ^8^Poniente Health Center, Córdoba-Guadalquivir Health District, Córdoba, Spain; ^9^Aeropuerto Health Center, Córdoba-Guadalquivir Health District, Córdoba, Spain; ^10^Cabra Health Center, Sanitary Management Area South of Córdoba, Córdoba, Spain; ^11^Group/Program Communication and Health, semFYC, Barcelona, Spain; ^12^Santa Rosa Health Center, Córdoba-Guadalquivir Health District, Córdoba, Spain

**Keywords:** obstructive sleep apnea, validity, reliability, questionnaire, primary care, Berlin questionnaire, home respiratory polygraphy, screening

## Abstract

**Background:**

The obstructive sleep apnea syndrome (OSA) is a highly prevalent condition. In Spain and other countries, only 5%–9% of patients with OSA have been diagnosed and treated. The lack of accessibility to diagnosis is considered the main cause of this situation through easy-to-use screening instruments, it is necessary to check their validity and reliability in the context where they are to be used.

**Objective:**

To validate the Spanish translation of the Berlin questionnaire for screening for moderate or severe OSA in patients aged 40 years or more detected in primary care.

**Methods:**

A descriptive observational study, with a first qualitative phase of transcultural adaptation to Spanish using the translation-back-translation method. Setting: primary care level of the Spanish National Health System. A total of 255 patients recruited from 7 healthcare centers completed the study. The Berlin questionnaire was administered to the recruited patients, and subsequently, a respiratory polygraphy was performed to confirm the diagnosis of OSA. The concurrent criterion validity of the questionnaire and its reliability in terms of internal consistency and reproducibility (intra-observer agreement) were analyzed.

**Results:**

The patients’ mean age was 54.76 years (SD: 6.57; 95% CI: 53.53–54.99), and 54.12% were men (95% CI: 47.96–60.27). We found that 61.57% (95% CI: 55.57–67.57) presented OSA (apnea-hypopnea index-AHI >5), and 45.5% (95% CI: 17.05–57.92) presented moderate or severe (AHI >15) OSA. The Berlin questionnaire, with a cut-off point of 4.5, showed a sensitivity of 76.77% (95% CI: 67.94–85.59), a specificity of 74.49% (95% CI: 65.35–83.63), a positive predictive value of 75.25% (95% CI: 66.34–84.16), a negative predictive value of 76.04% (95% CI: 66.98–85.10), and an area under the curve of 0.786 (95% CI: 0.721–0.851). Cronbach’s alpha coefficient was 0.730 (95% CI: 0.668–0.784), and the Kappa index was 0.739 (95% CI, 0.384–1.000).

**Conclusion:**

The Spanish adaptation of the Berlin questionnaire has good validity and reliability as a test for the diagnostic screening of moderate or severe OSA in patients aged 40 years or older. The findings of our study confirm that primary care physicians should use such screening tools to predict OSA.

## Introduction

Obstructive sleep apnea syndrome (OSA) is a multifactorial clinical condition whose pathophysiology is produced by the conjunction of anatomical, muscular, neurological factors and other factors not yet identified, causing a collapse of the upper airway as a result of an imbalance between forces that tend to close it and those that keep it open, resulting in multiple episodes of complete (apnea) or partial (hypopnea) obstruction of the upper airway. This obstruction leads to poor sleep quality and intermittent hypoxemia with vascular repercussions (1). OSA is a risk factor related to cardiovascular diseases (1–3), hypertension, and stroke (4, 5), so the frequent association with other comorbidities makes management more complex. The most frequent nocturnal symptoms are snoring, observed apneas, gasping or choking episodes, abnormal movements, diaphoresis, or frequent awakenings. In contrast, the usual daytime symptoms are excessive daytime sleepiness, nonrestorative sleep, tiredness, headache, irritability, or depression (3). The most important risk factors are age, male sex, and body mass index (BMI), and variables influencing their onset or aggravation are alcohol, tobacco, sedatives, hypnotics, barbiturates, and supine position (5).

OSA is a highly prevalent condition (1, 3, 6–8). Benjafield et al. (6), in their study indicate OSA prevalence in the general population is between 4% and 30%. The International Consensus Document on obstructive sleep apnea, indicates that in men over 40 years of age, the prevalence of OSA ranges between 48% and 73% (7). In Spain, between 3% and 6% of the population suffers from OSA, with a very severe condition between 24% and 26% of this (3). It affects 0.7%–3% of children aged between 4 and 5 years (8). In adult women, the prevalence of OSA is 2%–4%, with the ratio between men and women being 2-3/1, with a tendency to equalize after menopause. The prevalence of OSA increases with age, tripling in the elderly compared to the middle-aged population (8, 9).

In Spain and other countries, only 5%–9% of patients with OSA have been diagnosed and treated. The lack of accessibility to diagnosis is considered the main cause of this situation (10). Polysomnography (PSG) has been considered the gold standard for diagnosing OSA. However, PSG requires an infrastructure and human resources that not all centers of the health system can cover because it requires the nocturnal hospitalization of the subjects. One of the alternative methods to PSG to try to avoid these drawbacks is home respiratory polygraphy (HRP), a procedure that consists of nocturnal monitoring of oxygen saturation, oronasal airflow, and breathing movements; it can be performed at home by the patients themselves, with the convenient prior training of the patient. This tool has high accuracy, with a sensitivity of 93.3%–96.6%, a specificity of 82.9%–100%, and a positive predictive value of 96.5% for diagnosing OSA. In patients with a strong or moderate suspicion of OSA, HRP represents a procedure that can replace PSG. However, given its lower specificity, doubtful results, although few, should be confirmed by PSG (11, 12).

Currently, no curative treatment is available for OSA, but the mechanical problem (upper airway closure) can be solved with the use of continuous positive airway pressure (CPAP) devices (1, 3), which has been associated with decreased risk of falls (13), strokes, cardiovascular, morbidity and mortality (5), and with an improvement in health-related quality of life (14).

Given the high hidden morbidity of OSA, and the lack of accessibility to the diagnostic procedures usually used, OSA screening tools, such as the Berlin questionnaire (BQ) (12), could be useful tools for healthcare professionals in primary care settings (PC). To date, no validated version of the BQ is available in Spain, so more studies are needed to provide higher consistency to the results obtained.

The main aim of this study was to validate the Spanish translation of the BQ to detect moderate or severe OSA in a population aged 40 years or older residing in Spain. The specific objectives were: (a) to perform the transcultural adaptation of the BQ from English into Spanish, (b) to determine the criterion validity, and (c) to verify its reproducibility in terms and its internal consistency.

## Methodology

### Study design

Multicenter descriptive observational study to validate a measuring tool.

### Setting

The study has been carried out by the Andalusian Health System (SAS), part of the decentralized Spanish National Health System, which provides free universal health insurance to all Spanish citizens. The project was carried out in 7 primary care centers of the Córdoba-Guadalquivir Health District, located in the province of Córdoba, 5 of them urban and 2 rural.

### Participants

The selection criteria were: (a) inclusion criteria: patients aged 40 years or over, of both sexes, who attended the health center for any reason and gave informed consent. (b) Exclusion criteria: patients with a previous diagnosis of OSA or who, due to illness, cognitive status, or low level of studies, were not able to answer the questions of the BQ or to perform the HRP, or patients receiving hypnotics treatment or with chronic alcoholism problems.

### Sample size and selection

Based on results of previous studies (3, 12), and using the statistical program Epidat (Program for epidemiological data analysis. V. 4.2. Ministry of Health, Xunta de Galicia, Spain; Pan american Health Organization -PAHO-WHO-; CES University, Colombia), for a sensitivity of the test of 77%, a specificity of 44%, a ratio of non-ill/ill -probable OSA- of 0.624, an absolute accuracy of 10%, and for a 95% confidence level, the sample size needed for conducting our study was estimated to be 248 subjects, of whom 153 should be ill and 95 healthy. The final sample size was 255 subjects, 157 ill and 98 healthy. Subjects were recruited by consecutive sampling among those who attended the participating health centers and met the selection criteria.

### Interventions and measurements

The steps followed in the process of validating the BQ to Spanish were as follows:

1. Transcultural adaptation: The reverse translation (forward-translation and-backward-translation) methodology was used for the cross-cultural adaptation of questionnaires for use in clinical research, proposed by the WHO (15). Forward and backward-translations were performed, followed by a synthesis and cultural adaptation through a qualitative methodology. A Spanish translator proficient in the source language of the tool (English) performed the forward-translation of the questionnaire from English into Spanish. Then a second translator, native English and fluent Spanish speaker, blind to the original questionnaire, performed the backward-translation (16). Each translator scored (0 to 10) their difficulty in finding a conceptually equivalent expression between both languages for each question in the questionnaire. A group of experts, composed of the research team members, classified the elements according to the difficulty the first two translators had in finding a conceptually equivalent expression. It was considered necessary to perform a new translation and a reverse translation of the elements of serious difficulty.
2. Validation of the questionnaire BQ: In a second step, recruitment was conducted in the health centers opportunistically of the subjects meeting the selection criteria. A face-to-face interview was conducted with the subjects who agreed to participate in the study and signed the informed consent. In this interview, a form collecting sociodemographic data (age and sex), anthropomorphic data (weight and height, calculating the BMI), and the BQ was completed. Subsequently, each patient was individually trained in the management of HRP, for which an *in situ* simulation of the placement of all the device electrodes was performed. The instructor ensured that the patients had correctly assimilated all the information necessary for the device to be placed that same night, which must be returned the next day.

The polygraph used was Screen & Go (Sibelmed), with 6 channels (air flow, thoracoabdominal movements, snoring, body position, pulse, and oxygen saturation). The recording time for each study was 6 h. A total of 16 out of the 255 HRPs performed were not recorded correctly, so they were repeated the next day, obtaining valid values on this second occasion.

Polygraphic studies were automatically analyzed by Bitmelad polygraph software and reviewed by an expert researcher in sleep-disordered breathing following the criteria of the Spanish Society of Pulmonology and Thoracic Surgery (SEPAR). This collaborating researcher remained blind to the result of the questionnaire. Between 6 and 8 weeks, a collaborating researcher, other than the one who previously collected the data, randomly selected a subsample of 31 people from all subjects recruited for the study to evaluate the reproducibility or reliability of BQ in terms of the intra-observer concordance. This interview was conducted by telephone. Fieldwork began in June 2019, had to be discontinued in 2020–21 due to the COVID-19 pandemic, and resumed in January 2022, ending in December 2022.

### Variables and data sources

The independent variables of the study were age, sex, body mass index (BMI = weight in kg/height in square meters), and neck circumference (in centimeters). The dependent variables were the apnea-hypopnea index (AHI), defined as the number of apneas plus hypopneas per 1 h of polygraphy or polysomnographic study (3), and questions from the BQ (9). BQ is a widely used non-invasive screening tool for identifying subjects with a high probability of having OSA. It includes five items on snoring (category 1), three items on daytime sleepiness (category 2), and one item on history of hypertension (category 3). The overall score is determined from responses to all 3 categories, classifying patients as high risk of OSA when they have a positive score in 2 or more categories; otherwise, they are considered low risk.

### Statistical analysis

A descriptive analysis of the quantitative variables (calculating the arithmetic mean, the standard deviation, and the distribution limits) and the qualitative variables (tabulation and calculation of the absolute and relative frequencies for the different groups) was performed. The statistical parameters were expressed with their 95% confidence intervals (95% CI). Then, a bivariate analysis was performed for sex and degree of OSA, using the Pearson’s chi-square test, a *p*-value ≤0.05 was considered significant, using two-tailed tests. Statistical analyzes were performed using SPSS V.19 (IBM, United States).

Criterion validity parameters were calculated (concurrent criterial validity), namely sensitivity, specificity, positive predictive value (PPV), negative predictive value (NPV), positive likelihood ratio (LR^+^), and negative likelihood ratio (LR^−^), in addition to the prevalence or overall efficacy of the test, with their corresponding 95% CI. The AHI obtained by HRP was compared with the sum of the scores of the BQ, calculating the area under the ROC curve (AUC) and determining the optimal cut-off points, performing the sex-disaggregated analysis.

The internal consistency of the questionnaire was determined through Cronbach’s alpha coefficient, interpreting the results according to the Oviedo and Campo (17) criteria, which state that the minimum acceptable value for Cronbach’s alpha is 0.70; below that value the internal consistency of the scale used is low. For its part, the maximum expected value is 0.90; above this value it is considered that there is redundancy or duplication. The concordance between observers was evaluated through the Cohen’s Kappa coefficient (*K*), interpreting the agreement level according to the Landis and Koch scale, which establishes the following categories according to the value found: *K* < 0, poor agreement; *K*: 0.01–0.20, slight agreement; *K*: 0.21–0.40, fair agreement; *K*: 0.41–0.60, moderate agreement; *K*: 0.61–0.80, substantial agreement; and *K*: 0.81–1.00, almost perfect agreement (18).

### Ethical-legal aspects

The project has been approved by the Research Ethics Committee of the Reina Sofia Hospital of Córdoba (Act No. 279, ref. 3915) and obtained the authorization of the Management / Direction of the Córdoba and Guadalquivir Health District. The principles established in the Declaration of Helsinki, in the European Convention (Council of Europe) on Human Rights and Biomedicine, and the requirements established in Spanish law were respected. The study complied with the standards of good clinical practice (art. 34 RD 223/2004; EU Directive 2001/20/EC). The processing of the personal data of the subjects participating in the study was in accordance with the provisions of the European Data Protection Regulation and Organic Law 3/2018 on Personal Data Protection and guarantee of digital rights.

## Results

The cross-cultural adaptation was successful because 8 out of the 10 items of the BQ were equivalent, and only 2 items showed minor modifications without affecting the meaning of the question. Consequently, this questionnaire was used to analyze its validity and reliability.

Initial eligibility of 283 was evaluated, of which 255 were invited to participate, signing the informed consent. No patient was excluded or withdrew from the study (completion rate: 100%).

The patients had a mean age of 54.76 years (SD: 6.57; range: 40–60; 95% CI: 53.53–55.99), without statistical differences between men and women, men being 54.12% (95% CI: 47.96–60.27). Regarding the BMI, the mean was 31.17 kg/m^2^ (SD: 6.57; range: 19.53–60.18; 95% CI: 30.36–31.98), and the mean neck circumference was 38.33 cm (SD: 5.78; range: 24–53; 95% CI: 37.62–39.05), with significant differences by sex in neck circumference (*p* < 0.001) and BMI (*p* = 0.002).

A total of 61.57% (95% CI: 55.57–67.57) of the patients were diagnosed with OSA (AHI >5). Moderate/severe OSA (AHI >15) was found in 38.43% of subjects (Table 1).

**Table 1:** Prevalence of obstructive sleep apnea (OSA) and its level of severity, according to sex.

	Non-OSA AHI ≤5% (95% CI)	Mild OSA 5 <AHI ≤15% (95% CI)	Moderate OSA 15 <AHI ≤30% (95% CI)	Severe OSA AHI >30% (95% CI)
Men *n* = 117	32.61 (24.69–40.53)	17.39 (10.99–23.79)	26.09 (18.67–33.51)	23.91 (16.71–31.12)
Women *n* = 138	45.30 (36.15–54.45)	29.06 (20.71–37.41)	18.80 (11.62–25.99)	6.84 (2.20–11.48)
Total *n* = 255	38.43 (32.43–44.70)	22.74 (17.75–28.89)	22.74 (17.75–28.89)	16.08 (11.79–21.17)

Non-OSA: Pearson’s chi-square = 4.310; *p* = 0.038. Mild OSA: Pearson’s Chi-square = 4.906; *p* = 0.027. Moderate OSA: Pearson’s Chi-square = 1.191; *p* = 0.167. Severe OSA: Pearson’s Chi-square = 13.682; *p* < 0.001. AHI, apnea-hypopnea index. 95% CI: 95% confidence interval.

**Figure 1:**
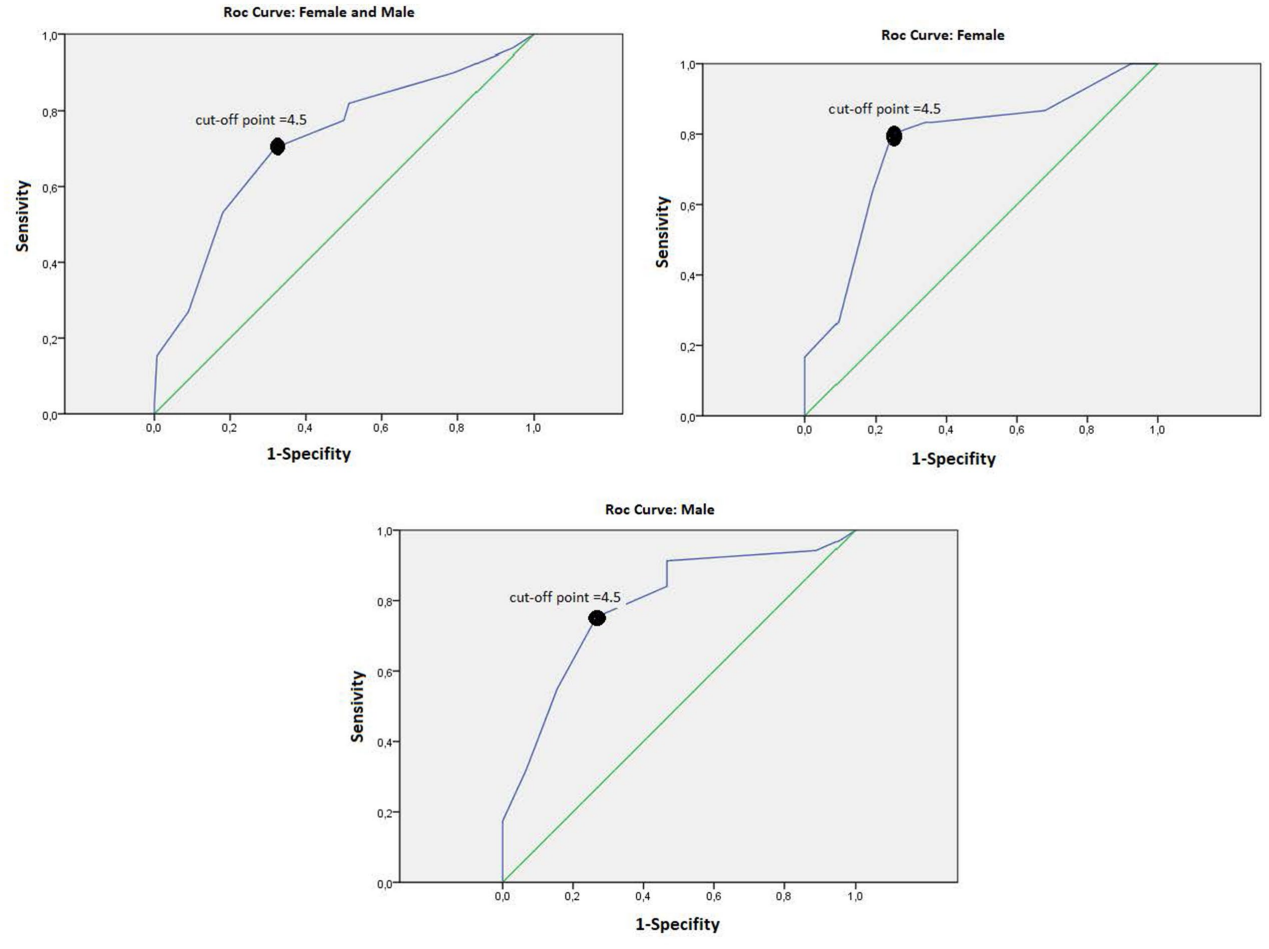
ROC curves of the Berlin questionnaire (cut-off point = 4.5).

Table 2 shows the results of criterion validity parameters according to sex. The BQ validated in Spanish for the population of 40 years or older, with a cut-off point of 4.5, presented a sensitivity of 76.77, a specificity of 74.49%, a positive predictive value of 75.25% (95% CI: 66.34–84.16), a negative predictive value of 76.04% (95% CI: 66.98–85.10), a LR^+^ of 3.01, and LR^−^ of 0.312. The AUC was 0.786 (95% CI: 0.721–0.851) (Figure 1).

**Table 2:** Criterion validity of the Berlin questionnaire for the detection of moderate or severe apnea syndrome [apnea-hypopnea index (AHI) >15], based on sex.

Parameters	Sex		Total *n* = 255
	Women *n* = 117	Men *n* = 138	
AUC value (95% CI)	0.773 (0.663–883)	0.786 (0.700–0.872)	0.786 (0.721–0.851)
Sensitivity % (95% CI)	80.00 (64.02–95.98)	75.36 (64.47–86.25)	76.77 (67.94–85.59)
Specificity % (95% CI)	75.47 (62.94–88.00)	73.33 (59.30–87.36)	74.49 (65.35–83.63)
PPV % (95% CI)	64.86 (48.13–81.60)	81.25 (70.91–91.59)	75.25 (66.34–84.16)
NPV % (95% CI)	86.96 (76.14–97.78)	66.00 (51.87–80.13)	76.04 (66.98–85.10)
LR^+^ % (95% CI)	3.26 (1.97–5.40)	2.84 (1.72–4.69)	3.01 (2.11–4.29)
LR^−^ % h (95% CI)	0.27 (0.13–0.55)	0.33 (0.21–0.52)	0.31 (0.21–0.45)
Prevalence % (95% CI)	36.14 (25.21–47.08)	60.87 (51.51–70.22)	50.25 (43.01–57.49)

AUC, area under the curve; PPV, positive predictive value; NPV, negative predictive value; LR^+^, positive likelihood ratio; LR^−^, negative likelihood ratio; 95% CI, 95% confidence interval.

Regarding the reliability of the questionnaire, Cronbach’s alpha coefficient was 0.730 (95% CI: 0.668–0.784), and the kappa index was 0.739 (95% CI: 0.384–1.000).

## Discussion

The BQ is an easy and quick to complete tool. From its origin, it has already proven to be of clinical utility as a screening tool because it has a high predictive capacity to detect OSA in those patients with high clinical suspicion of presenting this condition. This questionnaire resulted from the Sleep Conference, which brought together 120 American and German family practice physicians and sleep researchers in Berlin, Germany, in 1966 (12). Questions were selected from the literature to obtain factors or behaviors that, in all studies, systematically predicted the presence of sleep-disordered breathing. By consensus, the tool focused on a limited set of known risk factors for OSA. Its validation was conducted on 744 adults, of which only 100 underwent sleep studies, representing a methodological limitation. It presented a sensitivity of 86%, a specificity of 77%, and a positive predictive value of 89%. Questions about symptoms demonstrated good internal consistency (Cronbach’s alpha from 0.86 to 0.92) (12).

In the Spanish language, the validation of this questionnaire in the Colombian population detected in sleep centers was published in 2013 (19). This validation obtained a sensitivity of 87%, a specificity of 70%, a PPV of 98%, a NPV of 21%, and an AUC of 0.785, presenting an acceptable internal consistency with a Cronbach’s alpha coefficient of 0.725, and a high reproducibility, with a Cohen’s kappa index of 0.815. The authors found a clinically and statistically significant correlation between the adaptation performed and the PSG.

No validated version of the BQ for the Spanish adult population was available to date. Therefore, for cultural reasons or idiosyncrasies of each Spanish-speaking country (and probably also clinical or epidemiological reasons), it is necessary to ensure that it has the same qualities regarding its psychometric properties when applied to a population recruited in PC.

In the present study, we have successfully translated the BQ according to established guidelines. This study aligned with previous studies that examined sensitivity, specificity, PPV, and NPV. Table 3 shows that the sensitivity results are similar to the studies conducted in Greece (20), which are slightly higher than in Denmark (21). However, the specificity of our study is higher than that obtained in the rest of the published studies with which it has been compared.

**Table 3:** Validity and reliability of the Berlin questionnaire obtained in different published studies.

Studies	Netzer (12)	Polanía-Dussan (19)	Bouloukaki (20)	Lauritzen (21)	Present study
Place and year	Germany 1996	Colombia 2013	Greece 2013	Denmark 2018	Spain 2022
Type and number of patients studied	General primary care *n* = 744	General *n* = 212	General primary care *n* = 189	Sleep clinic *n* = 206	General. primary care *n* = 266
Used sleep test	PSG	PSG	PSG	CRM	HRM
Sex	NA	Men: 54.25% Woman: 45.75%	Men: 61.9% Woman: 38.1%	Men: 69.4% Woman: 30.6%	Men: 54.12% Woman: 45.88%
AUC	NA	0.785	0.59	NA	0.786
Sensitivity	86%	87%	76% (AHI ≥5 y <15) 84% (AH ≥15 y ≤30) 79% (AHI >30)	84%	76.77%
Specificity	77%	70%	40% (AHI ≥5 y <15) 61% (AHI ≥15 y ≤30) 39% (AHI >30)	17%	74.49%
VPP	89%	98%	80–94%	69%	75.25%
VPN	NA	21%	NA	33%	76.04%
Cronbach’s alpha coefficient	0.86–0.92	NA	NA	NA	0.730
Kappa index	0.68–0.98	0.7257	NA	NA	0.739

PSG, polysomnography; CRM, cardiac respiratory monitoring; HRP, home respiratory polygraphy; AUC, area under the curve; AHI, apnea-hypopnea index; PPV, positive predictive value; NPV, negative predictive value; NA, not available.

There are other questionnaires, such as the STOP-Bang (22–27) for the detection of OSA, that also show consistent results, as we have been able to confirm by administering it to the same sample of our population (23). This questionnaire showed a sensitivity not very different to BQ (range: 84.85%–93.8%), but a lower specificity (range: 55.10% to 63.4%) (23, 24, 26, 27), although presents the advantage of its greater usability, being even easier to fill in and interpret than the BQ.

## Conclusion

Our study demonstrates that BQ has a highly diagnostic capacity to detect clinically relevant OSA (AHI ≥15). BQ is simple and easy to understand and fill in, so it can be applied in a PC setting. The use of BQ could potentially identify many patients with OSA, especially when it is moderate or severe, leading to earlier correct diagnosis and faster treatment, decreasing the risk of complications and unwanted effects. With the translation, back-translation, and consensus of experts, a final version of the BQ applicable to Spain was obtained, which is understandable and usable, and retains the meaning of the questionnaire in its source language. This final version can be used for any Spanish patient over 40 years old, regardless of gender, educational level, and/or socioeconomic status. Extending the study with a PSG would be indicated only in the cases of a possible mild OSA. The results of this validation were similar to those of the source version, obtaining a qualified, affordable, and easy-to-use prediction tool in the PC setting with high diagnostic efficacy and acceptable reliability. This tool allows identifying patients with a higher risk of having OSA to schedule treatment more quickly and thus reduce the associated comorbidities.

### Strengths and limitations

The coronavirus disease (COVID-19) pandemic interrupted fieldwork, and although it was subsequently resumed, it has impacted the predetermined timelines, noticeably delaying the analysis and dissemination of results.

Although there may be a doubt that a selection bias could have occurred because we have not used probabilistic sampling techniques, it should be considered that convenience and consecutive sampling are usually used in this type of validation study. It has also been possible to produce a selection bias when including in the study patients who demand health care in health centers, since these may differ in terms of their personal or physical characteristics from those from the population who do not attend the centers primary care of the health system. It is possible that health professionals tended to include in the study those patients with symptoms suggestive of OSA, which would partly explain the high prevalence found. Likewise, and in consistency with what the stated by epidemiologist experts in methodology for validation of measuring tools (28), what is of primary importance is that the sample includes a wide range of subjects, from asymptomatic to patients with clear symptomatology of the studied disease, thus being represented the entire spectrum of the disease, so we understand that if there is a possible selection bias, this would be irrelevant.

Our study achieved the advantages of RP over polysomnography, especially in patients with a high probability of suffering from OSA. RP is a diagnostic modality of great interest because it represents a way of bringing the sleep study closer to the patient’s home and allows detection in a setting similar to that of the patient when he or she is about to sleep, without this being altered when performed in their natural environment and not in a setting, such as a hospital, which can cause dysfunctions and, as a consequence, increase the rate of false negatives or inconclusive results. This is supported by previous studies such as the one by Borsini et al. (29), where it is shown that the rate of study loss or equipment damage is low. Home PR, preceded by a meticulous instruction on the day the equipment is delivered, is a safe and reliable method. According to said study, 92.6% of the records met the pre-established quality criteria and allowed the diagnosis of moderate to severe OSA in a third of the population.

The blinded process used can be considered as a strength of the study, preventing the researcher who performed the polygraphic analysis from having prior knowledge of the results of the BQ, ensuring the decrease in committing a bias of information inherent to the psychological influence of knowledge of the interventions performed on the study participants.

## Data availability statement

The raw data supporting the conclusions of this article will be made available by the authors, without undue reservation.

## Ethics statement

The studies involving human participants were reviewed and approved by Comite de etica e investigacion clinica del hospital Reina Sofia, Córdoba, Spain. The patients/participants provided their written informed consent to participate in this study.

## Author contributions

RM-G, JS-M, LAP-dT, MV-A, and EN-M contributed to the conception and design of the study. JS-M, EN-M, and LAP-dT reviewed the database and performed the statistical analysis. EN-M wrote the first draft of the manuscript. All authors contributed to the article and approved the submitted version.

## Conflict of interest

The authors declare that the research was conducted in the absence of any commercial or financial relationships that could be construed as a potential conflict of interest.

## Publisher’s note

All claims expressed in this article are solely those of the authors and do not necessarily represent those of their affiliated organizations, or those of the publisher, the editors and the reviewers. Any product that may be evaluated in this article, or claim that may be made by its manufacturer, is not guaranteed or endorsed by the publisher.

## References

[1] Durán-Cantolla J Puertas-Cuesta FJ Pin-Arboledas G Santa María-Cano J. EGEDS (Ges). Documento de consenso nacional sobre el síndrome de apneas-hipopneas del sueño. Arch Bronconeumol. (2005) 32:1–14. doi: 10.1016/S0210-5705(09)71003-9

[2] Newman AB Nieto J Guidry U Lind BK Redline S Sharar E. Relation of sleep-disordered breathing to cardiovascular risk factors. The sleep heart health study. Am J Epidemiol. (2001) 154:50–9. doi: 10.1093/aje/154.1.50, PMID: 11434366

[3] Durán-Cantolla J Mar J De La Torre G Rubio R Guerra L. El síndrome de apneas-hipopneas durante el sueño (SAHS) en España. Disponibilidad de recursos para su diagnóstico y tratamiento en los hospitales del estado español. Arch Bronconeumol. (2004) 40:259–63. doi: 10.1016/s1579-2129(06)70096-9, PMID: 15161592

[4] Munoz R Duran-Cantolla J Martínez-Vila E Gallego J Rubio R Aizpuru F. Severe sleep apnea and risk of ischemic stroke in the elderly. Stroke. (2006) 37:2317–21. doi: 10.1161/01.STR.0000236560.15735.0f, PMID: 16888274

[5] Sahlin C Sandberg O Gustafson Y Bucht G Carlberg B Stenlund H. Obstructive sleep apnea is a risk factor for death in patients with stroke: a 10-year follow-up. Arch Intern Med. (2008) 168:297–301. doi: 10.1001/archinternmed.2007.70, PMID: 18268171

[6] Benjafield AV Ayas NT Eastwood PR Heinzer R Ip MSM Morrell MJ. Estimation of the global prevalence and burden of obstructive sleep apnoea: a literature-based analysis. Lancet Respir Med. (2019) 7:687–98. doi: 10.1016/S2213-2600(19)30198-5, PMID: 31300334 PMC7007763

[7] Mediano O González Mangado N Montserrat JM Alonso-Álvarez ML Almendros I Alonso-Fernández A. International consensus document on obstructive sleep apnea. Arch Bronconeumol. (2022) 58:52–68. doi: 10.1016/j.arbres.2021.03.017, PMID: 33875282

[8] Abrishami A Khajehdehi A Chung F. A systematic review of screening questionnaires for obstructive sleep apnea. Can J Anesth. (2010) 57:423–38. doi: 10.1007/s12630-010-9280-x, PMID: 20143278

[9] García Díaz EM Capote Gil F Cano Gómez S Sánchez Armengol A Carmona Bernal C Soto Campos JG. Poligrafía respiratoria en el diagnóstico del síndrome de apneas obstructivas durante el sueño. Arch Bronconeumol. (1997) 33:69–73. doi: 10.1016/S0300-2896(15)30656-6, PMID: 9091116

[10] Hidalgo-Martínez P Lobelo R. Epidemiología mundial, latinoamericana y colombiana y mortalidad del síndrome de apnea-hipopnea obstructiva del sueño (SAHOS). Rev Fac Med. (2017) 65:17–20. Available at: http://revistas.unal.edu.co/index.php/revfacmed/article/view/59565

[11] Lloberes P Durán-Cantolla J Martínez-García MÁ María Marín J Ferrer A Corral J. Diagnóstico y tratamiento del síndrome de apneas-hipopneas del sueño. Arch Bronconeumol. (2011) 47:143–56. doi: 10.1016/j.arbres.2011.01.001, PMID: 21398016

[12] Netzer NC Stoohs RA Netzer CM Clark K Strohl KP. Using the Berlin questionnaire to identify patients at risk for the sleep apnea syndrome. Ann Intern Med. (1999) 131:485–910. doi: 10.7326/0003-4819-131-7-199910050-00002, PMID: 10507956

[13] Onen F Higgins S Onen SH. Falling-asleep-related injured falls in the elderly. J Am Med Dir Assoc. (2009) 10:207–10. doi: 10.1016/j.jamda.2008.10.008 19233062

[14] Lizán Tudela L Reig FA. La evaluación de la calidad de vida relacionada con la salud en la consulta: las viñetas COOP/WONCA. Aten Primaria. (2002) 29:378–4. doi: 10.1016/S0212-6567(02)70587-8, PMID: 11996720 PMC7669103

[15] World Health Organization. Official WHO process of translation and adaptation of research instruments. (2016). Available at: http://www.who.int/substance_abuse/research_tools/translation/en/.

[16] Sousa VD Rojjanasrirat W. Translation, adaptation and validation of instruments or scales for use in cross-cultural health care research: a clear and user-friendly guideline. J Eval Clin Pract. (2011) 17:268–74. doi: 10.1111/j.1365-2753.2010.01434.x, PMID: 20874835

[17] Oviedo HC Campo-Arias A. Aproximación al uso del coeficiente alfa de Cronbach. Rev Colomb Psiquiatr. (2005) 34:572–80.

[18] Landis JR Koch GG. The measurement of observer agreement for categorical data. Biometrics. (1977) 33:159–74. doi: 10.2307/2529310, PMID: 843571

[19] Polanía-Dussan IG Escobar-Córdoba F Eslava-Schmalbach J Netzer NC. Validación colombiana del cuestionario de Berlín. Rev Fac Med. (2013) 61:231–8. Available at: https://repositorio.unal.edu.co/handle/unal/74444

[20] Bouloukaki I Komninos ID Mermigkis C Micheli K Komninou M Moniaki V. Translation and validation of Berlin questionnaire in primary health care in Greece. BMC Pulm Med. (2013) 13:6. doi: 10.1186/1471-2466-13-6 23347772 PMC3561101

[21] Lauritzen E Kørvel-Hanquist A Homøe P. The Danish translation and validation of the Berlin questionnaire for sleep apnoea. Dan Med J. (2018) 65:A5502. PMID: 30187861

[22] Serrano Merino J Pérula De Torres LA Muñoz Gómez R Roldán Villalobos A Feu Collado MN Ruiz-Moral R. Impact of CPAP therapy on health-related quality of life in elderly patients with apnoea-hypopnea syndrome: a systematic review of randomized clinical trials. Eur Respir J. (2017) 49:10–3. doi: 10.1183/13993003.01644-2016 28100549

[23] Muñoz-Gómez R Navarrete-Martínez E Serrano-Merino J Silva-Gil F Roldán-Villalobos A Martín-Rioboó E. The usefulness of the Spanish version of the STOP-Bang questionnaire for screening for moderate or severe sleep apnea syndrome in primary care. Front Public Health. (2022) 10:975114. doi: 10.3389/fpubh.2022.975114, PMID: 36159274 PMC9502031

[24] Chiu HY Chen PY Chuang LP Chen NH Tu YK Hsieh YJ. Diagnostic accuracy of the Berlin questionnaire, STOP-BANG, STOP, and Epworth sleepiness scale in detecting obstructive sleep apnea: a bivariate meta-analysis. Sleep Med Rev. (2017) 36:57–70. doi: 10.1016/j.smrv.2016.10.004, PMID: 27919588

[25] Amra B Rahmati B Soltaninejad F Feizi A. Screening questionnaires for obstructive sleep apnea: an updated systematic review. Oman Med J. (2018) 33:184–92. doi: 10.5001/omj.2018.36, PMID: 29896325 PMC5971053

[26] Cruces-Artero C Hervés-Beloso C Martín-Miguel V Hernaiz-Valero S Lago-Deibe FI Montero-Gumucio M. Precisión diagnóstica del cuestionario STOP-Bang sobre la apnea del sueño moderada en atención primaria. Gac Sanit. (2018) S0213-9111:30137–7. doi: 10.1016/j.gaceta.2018.05.003 30033095

[27] Cruces-Artero C Hervés-Beloso C Martín-Miguel V Hernaiz-Valero S Lago-Deibe FI Montero-Gumucio M. Utilidad diagnóstica del cuestionario STOP-Bang en la apnea del sueño moderada en atención primaria. Gac Sanit. (2019) 33:421–6. doi: 10.1016/j.gaceta.2018.05.003, PMID: 30033095

[28] Jenicek M Cléroux R. Epidemiología. Principios, técnicas, aplicaciones. Salvat; Barcelona. (1987) 13–32.

[29] Eduardo B Lorena M Tamara D Martín B Silvia Q Julio C. Estrategia de utilización domiciliaria de la poligrafía respiratoria con instalación por el propio paciente. Rev Am Med Respi. (2013) 13:04–11. Available at: http://www.scielo.org.ar/scielo.php?script=sci_arttext&pid=S1852-236X2013000100003&lng=es

[30] Pack AI. Sleep-disordered breathing: access is the issue. Am J Respir Crit Care Med. (2004) 169:666–7. doi: 10.1164/rccm.2401008, PMID: 15003949

